# Managing the changing burden of cancer in Asia

**DOI:** 10.1186/1741-7015-12-3

**Published:** 2014-01-08

**Authors:** Rengaswamy Sankaranarayanan, Kunnambath Ramadas, You-lin Qiao

**Affiliations:** 1 The International Agency for Research on Cancer, 150 Cours Albert Thomas, 69372, Lyon, Cedex 08, France; 2 Regional Cancer Centre, PO Box 2417, Trivandrum 695011, Kerala State, India; 3 Department of Cancer Epidemiology, Cancer Institute, Chinese Academy of Medical Sciences and Peking Union Medical College, Beijing 100021, China

**Keywords:** Asia, Cancer burden, Prevention, Screening, Early detection, Diagnosis, Treatment, Clinical implication, Health services, Survival

## Abstract

Asia accounts for 60% of the world population and half the global burden of cancer. The incidence of cancer cases is estimated to increase from 6.1 million in 2008 to 10.6 million in 2030, due to ageing and growing populations, lifestyle and socioeconomic changes. Striking variations in ethnicity, sociocultural practices, human development index, habits and dietary patterns are reflected in the burden and pattern of cancer in different regions. The existing and emerging cancer patterns and burden in different regions of Asia call for political recognition of cancer as an important public health problem and for balanced investments in public and professional awareness. Prevention as well as early detection of cancers leads to both better health outcomes and considerable savings in treatment costs. Cancer health services are still evolving, and require substantial investment to ensure equitable access to cancer care for all sections of the population. In this review, we discuss the changing burden of cancer in Asia, along with appropriate management strategies. Strategies should promote healthy ageing via healthy lifestyles, tobacco and alcohol control measures, hepatitis B virus (HBV) and human papillomavirus (HPV) vaccination, cancer screening services, and vertical investments in strengthening cancer healthcare infrastructure to improve equitable access to services.

## Introduction

Asia is the most diverse and populous continent; 4.3 billion of the world’s 7.1 billion people live there, and the population will increase by 1 billion by 2050 [1]. As a consequence of continuing socioeconomic development and increasing control of communicable diseases, life expectancy in all Asian countries has significantly increased. The proportion of people aged 65 years and above is likely to double from the current 7% by 2030. It is well known that cancer risk increases with age [2,3]. Changing lifestyles, increasing urbanisation, changes in reproductive patterns and diet, obesity, tobacco use, alcohol drinking, chronic infection and increasing lifespans contribute to an ever-increasing cancer burden and changing cancer pattern in Asian countries. The projected increase will be largest in low-resource and medium-resource countries in Asia. Excluding rich economies such as Japan, Hong Kong, Singapore, South Korea, Saudi Arabia, United Arab Emirates, Kuwait, Qatar and Bahrain, most Asian countries are still developing.

Not surprisingly, Asian countries have highly variable health services development and healthcare infrastructures as a consequence of their diverse economic development, healthcare policies and investments. Although the number of people living below poverty line fell by 430 million between 2005 and 2010, Asia is still home to half of the world’s extremely poor population. Thus, cancers associated with both lower and higher socioeconomic conditions contribute to the Asian cancer pattern and burden.

Asia is very heterogeneous, and although high-income countries such as Israel, Kuwait, Qatar, the Republic of Korea, Singapore and United Arab Emirates have well-developed health services, the vast majority of the population living in many low- and middle-income Asian countries (LMICs) have extremely limited services and the cancer burden is substantial in these countries. Despite the growing burden of cancer, it remains a low priority in healthcare planning and expenditure in most Asian LMICs. In this review, we discuss the changing burden of cancer in Asia, highlight the readily implementable control measures that, based on current evidence, can help reduce the burden of disease, and also discuss the health service reforms that are required to implement these strategies.

### Cancer patterns and burden in 2008 and 2030

Asia is a diverse continent. The striking variations in ethnicity, social norms, sociocultural practices and traditions, socioeconomic development and habits and dietary customs there are reflected in the patterns and burdens of cancer in different regions of Asia [2-4]. The estimated incident cases and deaths from the ten most common cancers in men and women in Asia around 2008 and projected for 2030 are given in Tables 1 and 2[3]. Incident cancer cases are estimated to increase in Asia from 6.1 million in 2008 to 10.7 million in 2030 and cancer deaths from 4.1 million in 2008 to 7.5 million in 2030 [3]. These projections mostly take into account demographic changes in the population. The disability-adjusted life years (DALYs) per 100,000 persons due to major cancers are given in Table 3[5]. The age-standardised rate of DALYs per 100,000 persons ranged between 1,748 and 2,786 in different regions of Asia and infection-related cancers (liver, stomach and cervix) contributed 27% of DALYs in East Asia [5].

**Table 1:** Incidence of and mortality from 10 most common cancers in Asia: men

**Cancer type**		**2008**			**2030**	
	**Cases, N**	**ASR (W) per 100,000**	**Deaths, N**	**ASR (W) per 100,000**	**Cases, N**	**Deaths, N**
Lung	604,629	32.4	523,899	28.1	1,139,685	1,003,977
Stomach	484,411	25.9	342,163	18.3	908,761	652,371
Liver	416,589	21.6	376,006	19.5	723,911	661,184
Colorectum	283,596	15.1	144,980	7.7	524,520	275,008
Oesophagus	247,060	13.2	204,919	11	458,775	389,596
Prostate	133,212	7.2	59,669	3.2	272,336	121,754
Bladder	101,776	5.4	44,316	2.4	193,191	88,480
Leukaemia	95,941	4.8	76,962	3.8	135,960	112,104
Lip, oral cavity	91,327	4.7	54,518	2.8	154,739	96,108
Non-Hodgkin lymphoma	75,866	3.9	50,707	2.6	123,173	85,260
All: Asia	3,241,249	170.6	2,353,611	124.2	5,824,230	4,324,238
All: World	6,617,844	202.8	4,219,626	127.9	11,471,506	7,422,358

Source: adapted from Ferlay *et al*. [3].

*ASR (W)*, age-standardised rate (standardised to world standard population).

**Table 2:** Incidence of and mortality from 10 most common cancers in Asia: women

**Cancer type**		**2008**			**2030**	
	**Cases, N**	**ASR (W) per 100,000**	**Deaths, N**	**ASR (W) per 100,000**	**Cases, N**	**Deaths, N**
Breast	528,927	26	193,497	9.5	818,220	321,106
Cervix uteri	312,990	15.3	159,894	7.9	473,001	267,180
Lung	268,434	13.1	229,778	11	512,745	446,879
Stomach	243,154	11.7	188,427	8.9	458,509	364,559
Colorectum	225,688	11	122,034	5.8	421,906	235,470
Liver	167,851	8.2	157,719	7.6	312,801	298,119
Corpus uteri	131,178	6.6	35,044	1.7	214,877	63,526
Oesophagus	124,507	6.1	103,313	5.0	237,659	201,705
Ovary	102,408	5.1	60,142	3.0	162,018	102,920
Leukaemia	76,111	3.8	60,298	3.0	108,189	87,708
All: Asia	2,851,110	139.6	1,718,721	83.2	4,854,983	3,127,901
All: World	6,044,710	164.4	3,345,176	87.2	9,790,012	5,661,912

Source: adapted from Ferlay *et al*. [3].

*ASR (W)*, age-standardised rate (standardised to world standard population).

**Table 3:** Age-adjusted disability-adjusted life year (DALY) rates per 100,000 population by cancer site and region, 2008

**Cancer type**	**East Asia**^**a**^	**China**	**Southeast Asia**	**South-central Asia**^**b**^	**India**
Oral cavity and other pharynx (C00 to C14, excluding C11)					
Male	103	23	71	198	319
Female	21	11	46	134	130
Oesophagus (C15)					
Male	119	328	49	144	123
Female	17	155	26	142	86
Stomach (C16)					
Male	357	552	172	205	102
Female	186	305	125	141	71
Colorectum (C18 to C21)					
Male	300	162	193	106	68
Female	207	139	182	104	63
Liver (C22)					
Male	412	797	457	80	57
Female	134	303	189	61	26
Lung (C33 and C34)					
Male	491	705	472	334	187
Female	201	376	223	126	55
Breast (C50)					
Female	368	223	468	485	362
Cervix (C53)					
Female	112	141	243	363	466
Non-Hodgkin lymphoma (C82 to C85, C96)					
Male	68	38	118	106	57
Female	42	26	83	69	37
All sites but skin (C00 to C97, excluding C44)					
Male	2,553	3,238	2,450	2,086	1,606
Female	1,853	2,300	2,491	2,499	1,900
Both sexes	2,197	2,786	2,470	2,289	1,748

^a^Excluding China.

^b^Excluding India.

Source: adapted from Soerjomataram *et al*. [5].

The distribution of major cancers in different regions of Asia is given in Table 4; the most common cancers are lung, stomach, liver, colon/rectum and oesophagus in men and breast, lung, stomach, colon/rectum and liver cancers in women [2,3,5]. Head and neck cancers, comprising oral, oropharynx and laryngopharynx cancers, are major contributors to the cancer burden in the Indian subcontinent (Tables 3 and 4).

**Table 4:** Five most common cancers in men and women in different regions of Asia

**Sex**	**Asian geographical region**			
	**West Asia**	**South Central Asia**	**South East Asia**	**East Asia**
Men	Lung, colon and rectum, bladder, prostate, stomach	Lung, oral cavity, pharynx, stomach, oesophagus	Lung, liver, stomach, colon and rectum, prostate	Lung, stomach, liver, colon and rectum, oesophagus
Women	Breast, colon and rectum, stomach	Cervix, breast, oral cavity, ovary, oesophagus	Breast, cervix, colon and rectum, lung, liver	Breast, lung, stomach, colon and rectum, liver

Source: adapted from Ferlay *et al*. [3].

**Figure 1:**
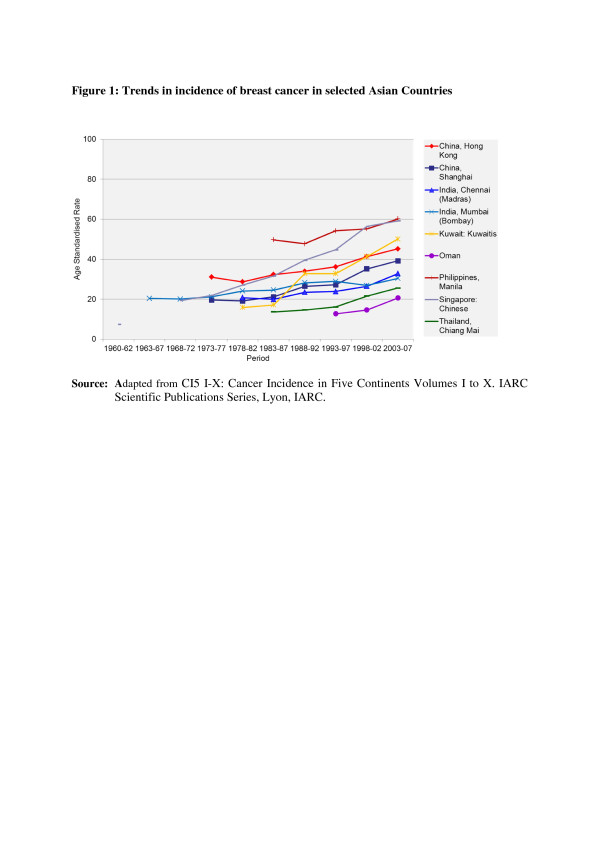
**Trends in the incidence of breast cancer in selected Asian countries.** CI5 I-X: Cancer Incidence in Five Continents Volumes I to X. IARC Scientific Publications Series, Lyon, IARC.

**Figure 2:**
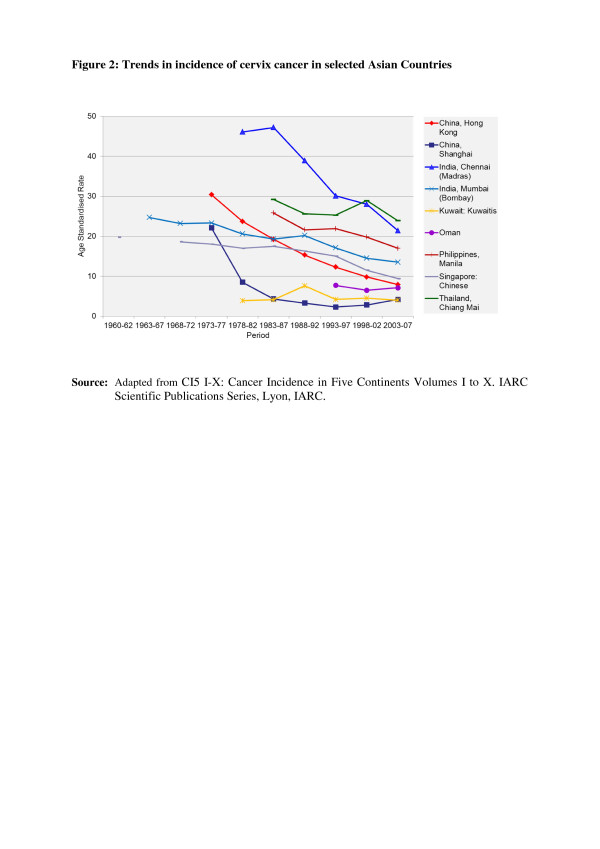
**Trends in the incidence of cervical cancer in selected Asian countries.** CI5 I-X: Cancer Incidence in Five Continents Volumes I to X. IARC Scientific Publications Series, Lyon, IARC.

**Figure 3:**
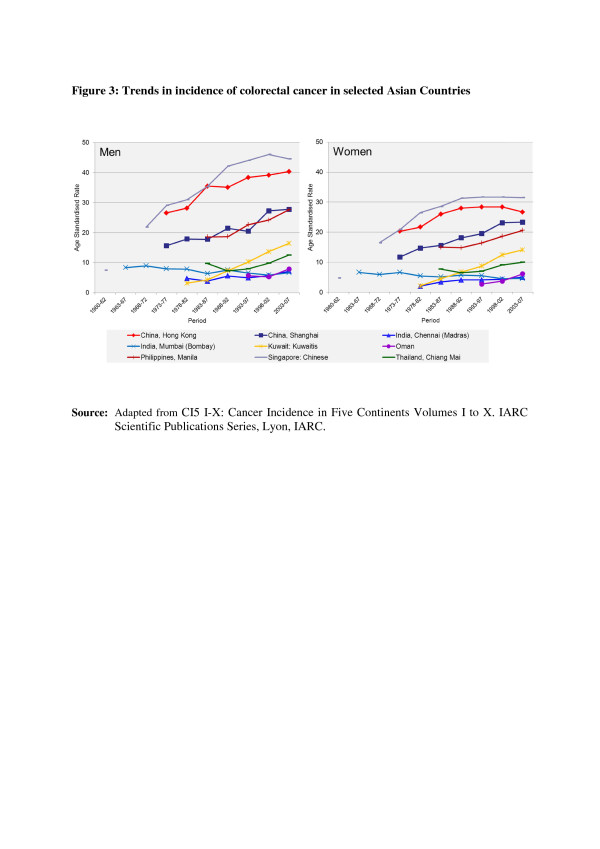
**Trends in the incidence of colorectal cancer in selected Asian countries.** CI5 I-X: Cancer Incidence in Five Continents Volumes I to X. IARC Scientific Publications Series, Lyon, IARC.

**Figure 4:**
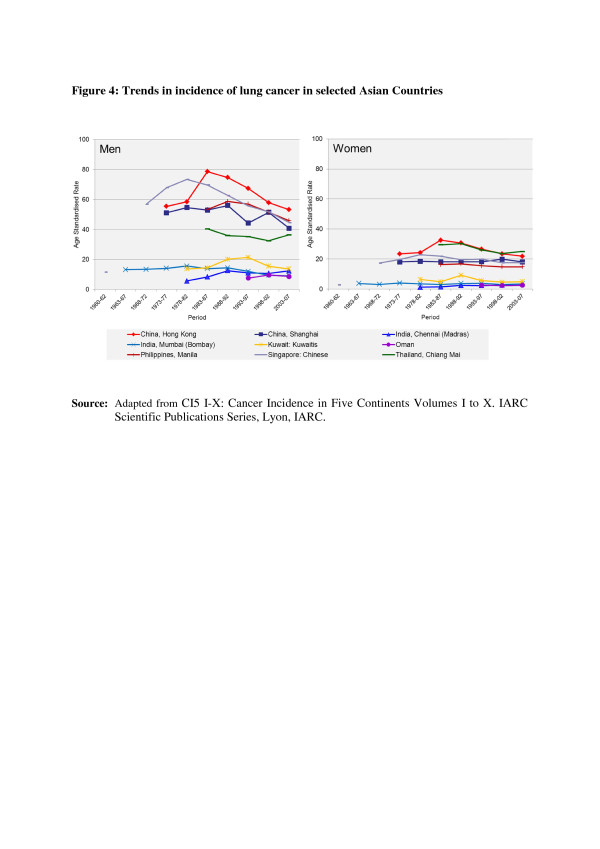
**Trends in the incidence of lung cancer in selected Asian countries.** CI5 I-X: Cancer Incidence in Five Continents Volumes I to X. IARC Scientific Publications Series, Lyon, IARC.

**Figure 5:**
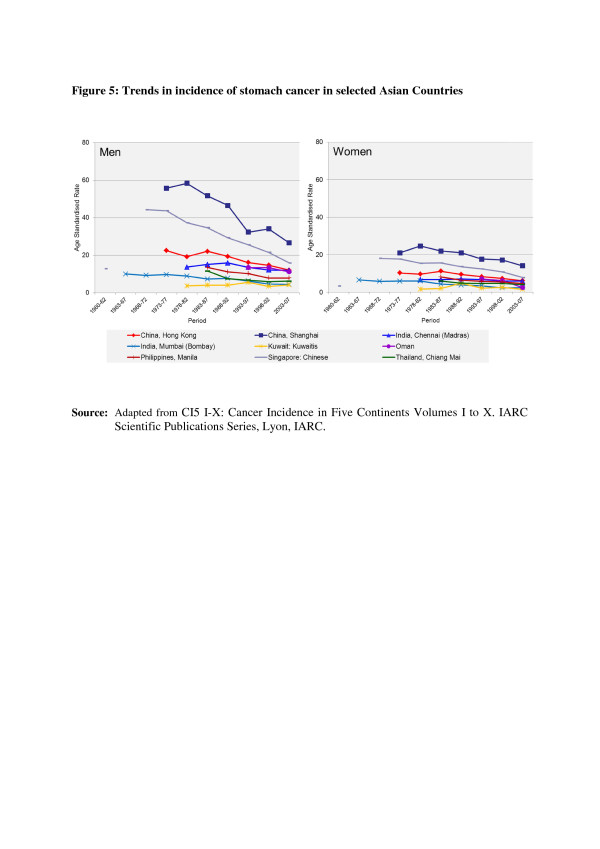
**Trends in the incidence of stomach cancer in selected Asian countries.** CI5 I-X: Cancer Incidence in Five Continents Volumes I to X. IARC Scientific Publications Series, Lyon, IARC.

**Figure 6:**
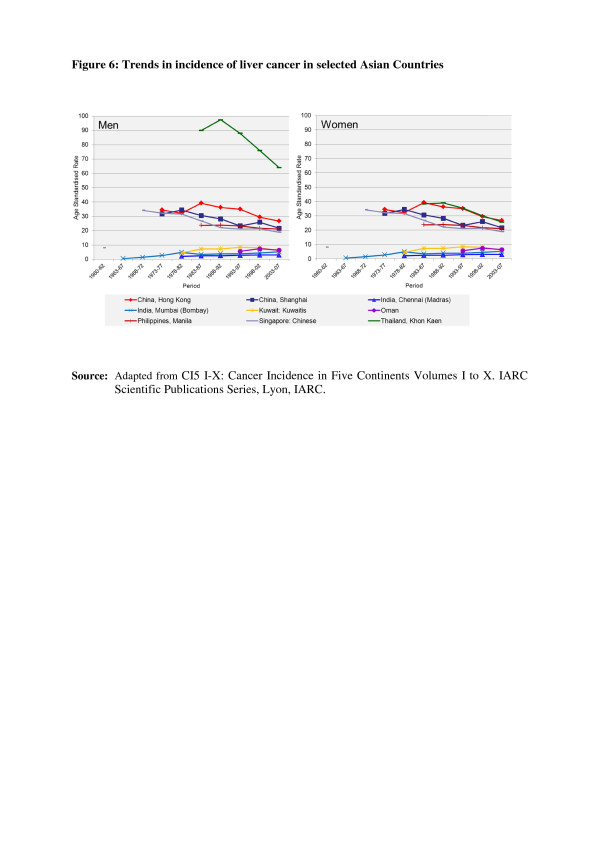
**Trends in the incidence of liver cancer in selected Asian countries.** CI5 I-X: Cancer Incidence in Five Continents Volumes I to X. IARC Scientific Publications Series, Lyon, IARC.

**Figure 7:**
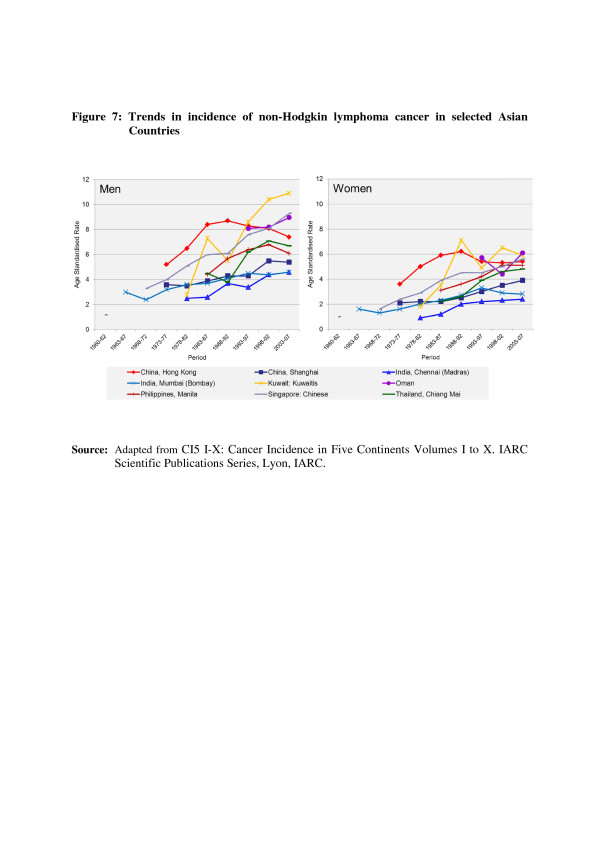
**Trends in the incidence of non-Hodgkin lymphoma in selected Asian countries.** CI5 I-X: Cancer Incidence in Five Continents Volumes I to X. IARC Scientific Publications Series, Lyon, IARC.

**Figure 8:**
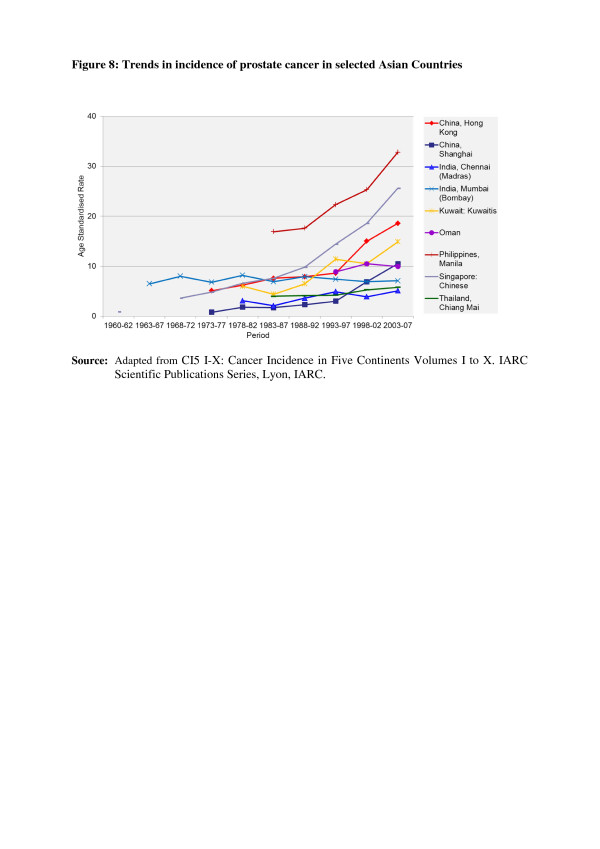
**Trends in the incidence of prostate cancer in selected Asian countries.** CI5 I-X: Cancer Incidence in Five Continents Volumes I to X. IARC Scientific Publications Series, Lyon, IARC.

In the countries of Asia with high human development index (HDI) scores, female breast, lung and colon/rectum cancers predominated, whereas in medium HDI countries oesophagus, stomach and liver cancers were also common; in low HDI regions cervical cancer was more common than breast cancer [6]. Incidence rates for breast, colorectal and thyroid cancers are higher among women high-income Asian countries due to the increasing adoption of Western lifestyles and overuse of ultrasound scanning of thyroid glands [6-9]. Whereas successful primary prevention and early detection initiatives for some cancers such as breast, cervix, oral cavity, lung, colon and rectum and so on may impact the future burden of these cancers, lack of or ineffective interventions may increase their burden over and above current projections. As per current trends, breast cancer incidence rates have been steadily increasing in all Asian countries, with an annual percentage increase of between 1% and 3% (Figure 1) [2,7,8]. Although cervical cancer incidence rates have been steadily declining or stable, the rates are still high in South and South-East Asia and a substantial number of Asian women continue to develop and die from the disease (Figure 2) [2,3]. Whereas colorectal cancer (CRC) incidence rates are increasing in both sexes in the Middle East, South East and East Asian countries, rates remain low in the Indian subcontinent (Figure 3) [7,9,10]. Trends in lung cancer incidence (Figure 4) closely follow population smoking prevalences, and rates are mostly stable or declining due to tobacco control measures [8]. Although stomach cancer incidence is steadily declining in both sexes (Figure 5), it is still the second most common cancer in men [5,8]. Liver cancer incidence rates are declining in South East and East Asian countries due to the effect of widespread hepatitis B virus (HBV) vaccination, reduced exposure to aflatoxins and improving living conditions (Figure 6) [11-16]. Oesophageal cancer incidence rates are steadily declining in high-incidence Asian countries due to better nutrition and improved fruit and vegetable intake [9,17]. Incidences of head and neck cancers are stable or declining due to tobacco control measures [8]. Among lymphoreticular malignancies, non-Hodgkin lymphoma (NHL) shows an increasing trend in incidence for reasons that are not entirely clear (Figure 7) [8]. Due to the ageing population, prostate cancer is on the increase (Figure 8) [8].

### Cancer healthcare services in Asian countries

The important components of cancer health services are given in Table 5. Cancer health services comprise policies and governance, integrated infrastructure and systems for awareness creation, prevention, early detection, referral, diagnosis, staging, treatment, follow-up care, palliative care and regular auditing of health services via monitoring and evaluation [18]. Health services should be supported by adequate financial resources through committed budget lines, and appropriate healthcare financing and cost recovery mechanisms for sustained delivery of services and information systems for monitoring and evaluation. Cancer health services are inadequate and still evolving in most Asian LMICs and a substantial variation in the development of the various cancer control components and access to cancer care can be seen between and within countries corresponding to their level of income and development (Table 6) [19,20]. For instance, all components of cancer care are well developed in high-income countries (HICs) such as Singapore and the Republic of Korea whereas services are underdeveloped in vast regions of many LMICs such as Bangladesh, Cambodia, China, India, Indonesia, Laos, Myanmar, Pakistan, Vietnam and Yemen, among several other countries (Table 6).

**Table 5:** Components of cancer health services

**Component**	**Factors**
Effective leadership and governance committed to health equity through sound public health policies and effective and accountable governance	Responsible for national cancer healthcare policies, plans and strategies and their implementation by effective governance of financing, infrastructure, human resources, drugs, technology and service delivery with relevant guidelines, plans and targets
Adequate financing of health services (health financing) to develop optimal healthcare infrastructure, recruitment and retention of human resources and to ensure universal health coverage by removing financial barriers to access and by preventing financial hardship and out-of-pocket catastrophic expenditure	Government budget lines, a system to raise and pool donor funds fairly
	Social security and employee insurance schemes and cost recovery mechanisms
	A financing governance system supported by relevant legislation, auditing and public expenditure reviews and clear operational rules to ensure timely and efficient use of funds
Adequate human resources for healthcare administration and delivery	Investing in and improving education through academic initiatives
	Recruitment, distribution, and retention by appropriate payment systems with right incentives
	Enhancing productivity, performance, competency and skills by in-service training, reorientation, continuing education opportunities, establishment of job-related norms, support systems, enabling work environments and job promotion opportunities
Ensuring universal access to essential diagnostics, vaccines, drugs and technologies	National lists of essential medical products, national diagnostic and treatment protocols, and standardised equipment per levels of care to guide procurement, to promote rational prescription and reimbursement
	A supply and distribution system to ensure universal access to essential medical products and health technologies through public and private channels, with focus on the poor and disadvantaged
	A medical products regulatory system for marketing authorisation, quality assurance and price and safety monitoring, supported by relevant legislation and enforcement mechanisms
Service delivery through a network of primary, secondary and tertiary care networks	Preventive services (health education, awareness, control of tobacco/alcohol/other cancer risk factors, healthy diet, promotion of physical activity, obesity/overweight control, hepatitis B virus (HBV) and human papillomavirus (HPV) vaccination)
	Early detection services (population awareness on early symptoms/signs, improving early detection skills of primary care practitioners by in-service training and reorientation, screening, early diagnosis, development of referral pathways)
	Diagnosis and staging (histopathology, cytology, haematology, immunohistochemistry, tumour markers, biochemistry, microbiology, x-ray, magnetic, ultrasound and nuclear imaging and endoscopy services)
	Treatment services (cancer surgery, radiotherapy, chemotherapy, hormone therapy targeted therapy, bone marrow transplantation, rehabilitation and counselling services), palliative care (oral morphine, other opioids and analgesics, adjuvant drugs, symptomatic treatments)
	Systems and establishments to render the above services (comprehensive cancer centres, specialised centralised services such as paediatric oncology services, oncology units in district and provincial hospitals, community cancer centres, cancer screening units, rural extension services for follow-up care in remote areas, palliative care units, palliative care teams, home care and community palliative care networks)
Health information initiatives and systems such as risk factor surveys, population based cancer registries, hospital cancer registries, medical records departments, screening programme and health insurance databases and death registers	To quantify cancer burden to facilitate planning cancer services
	To evaluate effectiveness of cancer control activities by monitoring trends in risk factor prevalence, trends in cancer incidence, stage distribution, survival and mortality

Adapted from World Health Organization key components of well functioning health systems (http://www.who.int/healthsystems/EN_HSSkeycomponents.pdf) and World Health Organization [18].

**Table 6:** Cancer health services in Asia by per capita gross national income (GNI, 2012) categories

**Income category**	**Countries (N = 48)**	**Cancer health services and infrastructure**
Low-income countries (per capita GNI < US$1,036 )	Afghanistan, Bangladesh, Cambodia, Democratic Republic of Korea, Kyrgyzstan Republic, Myanmar, Nepal, Tajikistan	Poorly developed healthcare infrastructure and overextended services far exceeding capacity, limited human resources, poorly supported by government financial resources. Healthcare financing is mostly by catastrophic out-of-pocket expenditure. The level of development and planned annual vertical investments by governments in infrastructure and in terms of financial and human resources fall far short of the level to ensure equitable access to preventive, diagnostic, treatment and follow-up care for the general population. More than three-quarters of patients with cancer do not receive adequate care, with poor survival prospects. Some countries such as Bangladesh are working towards universal health coverage.
Lower-middle-income countries (per capita GNI US$1,036 to US$4,085)	Armenia, Bhutan, India, Indonesia, Laos, Mongolia, Pakistan, Philippines, Sri Lanka, Syria, Timor-Leste, Uzbekistan, Vietnam, Yemen, West Bank and Gaza	Cancer health systems are fragmented and mostly centred in urban areas, with underinvestment in equipment, essential consumables and drugs and human resources development; vast regional variation of services within countries exists, with extremely limited availability of and access to care for rural and socioeconomically disadvantaged populations. Some countries such as India, Indonesia, Philippines, Sri Lanka and Vietnam are working towards universal health coverage.
Higher-middle-income countries (per capita GNI US$4,086 to US$12,615)	Azerbaijan, China, Georgia, Iran, Iraq, Jordan, Kazakhstan, Lebanon, Malaysia, Maldives, Thailand, Turkey, Turkmenistan	Cancer health systems are still evolving with less integrated multiple independent systems of care; considerable potential for further improvements in infrastructure, coverage and healthcare financing in most countries. Rural areas have inadequate services in large countries such as China. Some countries such as Thailand, Malaysia and Turkey have much better facilities and systems developed with universal health coverage providing seamless access for prevention, early detection and satisfactory clinical management of common cancers and improved survival outcomes.
High-income countries/regions (per capita GNI > US$12,616)	Bahrain, Brunei Darussalam, Hong Kong SAR of China, Israel, Japan, Republic of Korea, Kuwait, Macao SAR of China, Oman, Qatar, Saudi Arabia, Singapore, Taiwan, United Arab Emirates	High government investment in well organised healthcare infrastructure, well resourced and highly accessible diagnostic and treatment services, facilities exist for early detection, advanced state-of-the-art diagnostic and treatment services within public health services, expatriates contribute to a high proportion of human resources (healthcare providers) in West Asian high-income countries.

Sources: adapted from World Bank (http://www.worldbank.org/)/Sankaranarayanan *et al*. [20]/United Nations [23].

In general terms, cancer health services in all low-income and low-middle-income Asian countries are not adequately developed to deal with the increasing demands of preventive, diagnostic, therapeutic and follow-up care interventions needed to control the growing cancer epidemic in Asia. There are few facilities for cancer prevention, diagnosis and treatment, with weak referral systems, limited access to surgery, radiotherapy and essential cancer chemotherapeutic drugs in many low-income and middle-income Asian countries. Awareness among the general population and primary healthcare personnel about cancer, its symptoms and signs and improved treatment outcomes, if detected early, are conspicuously limited [21].

Although national cancer programmes, as outlined by the World Health Organization (WHO) [22], provide a framework for the development of cancer control services, there is a lack of cohesive national plans, inadequate financial and human resources and advocacy to develop and scale up comprehensive cancer prevention, early detection and treatment services. Less than 4% of gross domestic product (GDP) is spent on healthcare and government health expenditure per capita is less than 100 international dollars (1 international dollar is comparable to what can be purchased with US$1) in most low-income Asian countries, whereas this exceeds 2,200 international dollars in high-income Asian countries. The share of GDP spent on healthcare remained constant in most Asian countries, but declined in Afghanistan, Armenia, Bhutan, India, Maldives, Mongolia, Turkmenistan and Uzbekistan between 2000 to 2009 [23]. More than 60% of the total healthcare expenditure in most low-income and lower-middle-income Asian countries comes from private resources and more than 80% of private health expenditure is direct out-of-pocket payments, with catastrophic results on most families in these countries [23]. In 20 Asian countries, the health workforce density (includes doctors, nurses, midwives, multipurpose health workers and technicians) for the entire public health service is below the threshold of 23 workers per 10,000 population, a level below which essential health services are hampered; Cambodia has the lowest density at 10 health workers per 10,000 people [23]. As a consequence of the lack of cohesive national cancer plans and competition for resources within the limited overall health resources, cancer health services are not well funded by public and government finances and require high out-of-pocket payment in most low-income and lower-middle-income and some lower-middle-income Asian countries such as China and India. These countries exhibit substantial deficiencies in access to care for rural and disadvantaged populations, as evidenced by worse survival outcomes for patients living non-metropolitan and rural areas [19,20]. Migration of skilled healthcare workers, medical tourism and the increasing number of public hospitals functioning with corporate principles and private participation pose further challenges to the already strained cancer healthcare services in many Asian countries. Striking a balance between investment in preventive, early detection, diagnosis and treatment services and, allocation of resources among different treatment options, are additional challenges in cancer services.

Cancer health services, in terms of access to clinical diagnosis, endoscopy, imaging, cytology, histopathology, tumour markers, surgery, radiotherapy, chemotherapy, hormone therapy, palliative care and rehabilitation, as well as availability of trained human resources in cancer care, remain to be strengthened in a number of low-income and low-middle-income Asian countries [24].

Cancer staging at diagnosis, which describes the extent of disease based on the size and the degree to which it has spread to lymph nodes and distant organs, is a key factor in determining treatment and prognosis. The availability of imaging, endoscopy and histological assessment in health services is critical to accurately stage cancer. Beyond staging’s key role in clinical practice, the availability of population-level cancer stage data for major cancers such as breast, cervix, colorectum and head and neck adds value to outcomes such as survival and mortality. All Asian countries should improve facilities for cancer staging and make information available that will help to monitor the performance of the health system and patterns of care that may signal opportunities for improvements.

Cancer treatment is resource intensive and often multimodal, involving surgery, radiotherapy or both for the treatment of localised and advanced disease with systematic therapy. Development and implementation of evidence-based treatment guidelines and follow-up care for major cancers (for example, breast, colon, cervix) may be encouraged for optimum use of treatment resources in LMICs.

To generate the human resources essential to provide cancer control services in Asian countries requires long-term planning and investment in educational institutions. Since a large proportion of Asian healthcare workers migrate, such planning is even more critical.

### Opportunities for cancer prevention to reduce cancer occurrence in Asia

Substantial reduction in the incidence of lung and head and neck cancers and other tobacco-associated cancers can be achieved if tobacco control measures are further strengthened, particularly in low-income and low-middle-income Asian countries. Pricing and taxation, weaning of tobacco farmers and workers on to other occupations, smoke-free environments, banning both direct and indirect promotion of tobacco-containing products and health education are important components in a multipronged, multisectoral tobacco-control activity. The extent to which these tobacco control interventions are implemented varies widely across Asian countries. More earnest tobacco control implementation could substantially reduce the tobacco-related cancer burden in Asia [25-27].

The striking geographic variability in liver cancer incidence in Asia is largely explained by the distribution and natural history of HBV and hepatitis C virus (HCV) infections. Protection from vaccination lasts at least 20 years [28]. Vaccinating all infants against HBV, preventing HCV infection through blood screening for HCV antibodies, universal precautions against blood contamination in healthcare settings, reducing HCV transmission from injection drug use, effective antiviral therapy to control HBV infection or eradicate HCV, reducing exposure to aflatoxin B1 (AFB), alcohol control and control of chronic liver fluke infection are the major approaches for liver cancer prevention and control. The above control measures have led to a decline in liver cancer incidence in China, Hong Kong, Singapore, Taiwan and Thailand [11,13-16]. The hepatitis B vaccine is 95% effective in preventing HBV infection and sustaining a high coverage (including the birth dose in high-risk countries) through expanded immunisation programmes is vital to ensure future steady declines in liver cancer incidence.

Around 70% to 75% of cervical cancers are caused by human papillomavirus (HPV) 16 or 18 [29], and the currently available prophylactic HPV vaccines are highly effective in preventing the persistent infections and precancerous lesions caused by HPV [30] when given in early adolescence or adulthood. The HPV vaccine is highly effective in uninfected or HPV-naïve girls and women [30]. The safety and feasibility of vaccinating girls through existing health services for adolescent populations has been well established [31]. HPV vaccination is not yet implemented as part of national immunisation programmes in Asian countries other than in Bhutan and Malaysia. The low priority accorded to cervical cancer prevention by national planners, high cost of vaccines, service delivery challenges in vaccinating adolescent girls and antivaccination propaganda, as well as erroneous and false attribution of unrelated adverse events to vaccination in some countries, have proved to be major impediments in implementing HPV vaccination through national immunisation programmes in high-risk Asian countries [31]. Currently Bhutan and Malaysia are the only two Asian countries vaccinating 12-year-old girls through their national immunisation programmes where full dose coverage exceeds 95% [31,32]. HPV vaccination should be considered as a priority in high-risk Asian countries such as India and Thailand, among others, where early implementation will lead to a cohort of women at low risk for cancer in a few years’ time; this is even more relevant given the constraints in introducing organised screening programmes.

Eradication of *Helicobacter pylori* infection results in the healing of gastritis and reversal of early mucosal damage, and has the potential to prevent stomach cancer [33]. A test-and-treat approach to *H. pylori* in younger people and a combination of its eradication and endoscopic surveillance at periodic intervals for those aged 50 years and above has recently been proposed to eradicate stomach cancer [34]. Given the high prevalence of infection (over 60%), antibacterial treatment for all people who are chronically infected is impractical and may lead to antimicrobial resistance, and such interventions are not yet ready to be used in wider public health programmes. Given the increasing adoption of sedentary Western lifestyles in many Asian countries, primary prevention measures focusing on promoting healthy lifestyles such as increased physical activity, increased consumption of fresh vegetables and fruits, healthy diets, and decreased use of salted and preserved food through awareness programmes are important in Asian countries to reduce the risk of common cancers such as stomach, oesophagus, colon/rectum and breast, among other epithelial cancers.

### Role of screening in reducing cancer burden in Asia

Although screening has been shown to reduce both incidence and mortality of cervical and colorectal cancer and mortality from breast cancer, large-scale population-based screening programmes for the above cancers do not exist in Asian countries except in Japan, Hong Kong, South Korea, Taiwan and Thailand [27,35-39]. For screening to be effective, high quality interventions should be provided and accessed by a large proportion of the target population. This requires considerable organisation, diagnostic and treatment infrastructure and resources within routine health services. However, organised cancer screening programmes will not be feasible in the low-income and lower-middle-income countries of Asia for several years to come, given their limited healthcare infrastructures and resources.

Given the challenges in implementing quality-assured cytology screening, HPV testing and visual inspection with acetic acid (VIA) have been evaluated as alternative screening methods in study settings in China, India, Indonesia, Philippines and Thailand [36,40-46] and significant mortality reductions following a single HPV or VIA screen [43,45] have been demonstrated in randomised trials. Large-scale opportunistic screening with VIA is ongoing in routine health services nationally or regionally in Bangladesh [47], India [40], Indonesia [48] and Thailand [49]. Since screening programmes are not common, facilities for diagnosing and treating high-grade cervical intraepithelial neoplasia (CIN) using techniques such as colposcopy, cryotherapy, cold coagulation and loop electrosurgical excision procedure (LEEP) are extremely limited in many regions of Asia. Integrating implementation of HPV vaccination of 9 to 13-year-old girls and providing cervical screening commencing at age 30, preferably with HPV testing (at least a single screen or repeated at 10-year intervals for two to three rounds) is a pragmatic way of providing population-based cervical cancer prevention services in low-income and middle-income Asian countries. HPV vaccination will eventually result in a cohort of women at very low risk of cervical cancer, while the low-intensity screening will provide prevention services for older women not covered or protected by vaccination.

In high-income Asian countries such as Singapore, South Korea and Hong Kong, where widespread mammography screening and improved access to recent advances in treatment are available, more than 70% of breast cancers are diagnosed at stages 1 and 2, and overall breast cancer survival rates exceed 85% [19,20,27,50,51]. However, population-based mammography screening is neither feasible or cost effective in most Asian countries, given the considerable healthcare resources required for providing mammography and reporting and investigation of screen-positive women, and the resulting increased diagnostic and treatment demands [52,53]. The efficacy of organised clinical breast examination (CBE) screening programmes involving asymptomatic women in reducing breast cancer mortality is not yet known [53,54]. It was clear that the demands of diagnosis following CBE screening could not be met in a CBE screening trial in the Philippines, which is a lower-middle-income country, due to difficulties in access, and the study was abandoned [55]. It is not advisable to organise routine population-based organised CBE screening programmes until its effectiveness over and above routine healthcare has been established in randomised trials, and health service accessibility has improved [53,54]. However, opportunistic CBE and prompt referral of women with suspected breast lumps and other signs should be provided in primary care clinics in all Asian countries. The impact of such initiatives should be documented by trends in breast cancer stages in hospitals.

The current high-risk Asian countries for CRC are high-income countries with well-developed health services that can sustain organised screening based on faecal occult blood testing (FOBT) and colonoscopy triage of screen-positive persons. Widespread FOBT screening and colonoscopy triage of screen-positive individuals has been on going in Japan, Singapore, South Korea and Hong Kong over the last two decades. CRC mortality has started declining, despite increasing incidence in these countries, as a consequence of improved early detection through screening and improved access to surgery and adjuvant chemotherapy [56]. However, further efforts should be made for these programmes to fully evolve with satisfactory coverage and inputs that can ensure further substantial reductions in CRC incidence in high-risk countries. Recently, the feasibility of introducing CRC screening was demonstrated in a pilot programme in Lampang province in Thailand [57].

Oral precancerous lesions and early preclinical oral cancers can be detected by oral visual screening by doctors, nurses and health workers. Oral visual screening has been shown to be effective and cost effective in reducing oral cancer mortality among users of tobacco and/or alcohol in a randomised trial in India, supporting the introduction of population-based oral cancer screening in high-risk individuals in high-incidence countries [40,58,59].

High-risk Asian countries such as the Republic of Korea and Japan have introduced large-scale stomach cancer screening programmes, despite the paucity of evidence to support stomach cancer screening in the general population. In an analysis of around 2.7 million persons who underwent either upper gastrointestinal series (UGIS) radiological imaging or endoscopy screening, the positivity rates were 4.0% and 4.3% respectively; the interval cancer rates within 1 year of a negative screen were similar (1.2/1,000 negative screens) with both screening approaches. The sensitivity of UGIS and endoscopy in detecting stomach cancer was 36.7% and 69.0% respectively, indicating endoscopy performed better overall than UGIS [60]. However, data on the impact of endoscopy screening on gastric cancer mortality is lacking, and the assessment of the utility of such screening approaches should take into account the feasibility, costs involved and reduction in mortality.

Population-based screening for lung cancer with spiral computed tomography (CT) is not recommended for Asian countries, given the uncertainty about harms from screening and the generalisability the results of the Western randomised trials, and the cumbersome, non-feasible screening methods [61] for stomach (barium meal photofluorography, serum pepsinogen estimation, endoscopy), oesophagus (cytology-based on non-endoscopic cell sampling methods, endoscopy) and liver (serum α-fetoprotein, ultrasonography) cancer are not recommended due to lack of efficacy data, limited access to endoscopy equipment and limited expertise in mass screening [62-64]. Prostate cancer testing using prostate-specific antigen (PSA) is not recommended for Asian countries due to the significant harms associated with overdiagnosis and overtreatment, the inability of the PSA test to differentiate between indolent and lethal prostate cancer, and lack of mortality reduction following screening [65].

### Early diagnosis and appropriate treatment to improve survival outcomes

Cancer health services in terms of access to clinical care, referral, endoscopy, histopathology, surgery, radiotherapy, chemotherapy and palliative care should be strengthened in all low-income and middle-income countries to provide basic diagnosis and treatment services for patients with cancer. More than 70% of cancers in low-income and middle-income Asian countries are now diagnosed in locally advanced clinical stages, with overall 5-year survival being less than 50% [19,20]. Low awareness, delay in seeking diagnosis after symptoms, advanced clinical stages and a significant proportion of patients not accessing care or not completing treatment, due to poorly developed and poorly accessible healthcare services or unaffordability due to socioeconomic factors, are principal reasons for the observed low cancer survival in low-income and middle-income Asian countries [19,20,66]. However, the high survival for many cancers observed in Japan, Singapore, South Korea, Hong Kong SAR and Taiwan reflect the impact of better organised and developed cancer health services. Therefore, it is apparent that improving cancer awareness, improving access to adequate health services, early detection and prompt treatment are the major public health and clinical approaches to improve survival and to prevent premature deaths from cancer.

Early stage breast cancers with small tumours (5 cm or less, T1 or T2 disease) and absence of axillary lymph node metastasis (N0 disease) and the recent advances in locoregional and systemic adjuvant treatments are associated with improved long-term survival [19,20,67]. Low breast cancer awareness, inadequate pathology services for diagnosis and staging and fragmented treatment were identified as the biggest challenges in breast cancer control in low-income countries in a recent consensus paper from the Breast Health Global Initiative [66]. Given the increasing burden of breast cancer in Asia, investments must be made in public healthcare infrastructures. These investments should ideally improve breast cancer awareness, ensure prompt referrals from primary care and rapid diagnosis and treatment. Healthcare services should have adequate facilities for triple diagnosis involving clinical breast examination, diagnostic imaging (mammography and/or ultrasonography) and fine needle aspiration cytology (FNAC) or excision biopsy, histopathology services, including testing for hormone receptors by immunohistochemistry, and locoregional and systemic treatment by surgery, radiotherapy and adjuvant chemotherapies, hormone therapies, and follow-up care [66]. All women with invasive breast cancer in all Asian countries should be tested for oestrogen receptor (ER) and progesterone receptor (PR) status in order that they can have therapies tailored to them. For instance, women with ER-positive breast cancers have a better prognosis and are candidates for hormone therapy [66,68]. Improved breast cancer awareness and access to effective diagnosis and treatment played an important role in improving early diagnosis and survival in high-income countries before the introduction of widespread mammography screening [69-71]. Improving surgical and radiotherapy facilities and the feasibility to administer systemic therapies in health services will increase breast cancer survival significantly in many Asian countries [19,20,66].

Surgery is the mainstay in the treatment of patients with stage 0 to 1 CRC, whereas both surgery and adjuvant chemotherapy are indicated in the management of stage 2 and 3 CRC. Treatment of early clinical stages is associated with excellent prognosis: 5-year survival for stages 1 and 2 CRC exceed 90% and 80%, respectively and ranges between 30% to 60% for stage 3 and less than 10% for stage 4 in Japan [72,73]. However, 5-year survival rates are much lower in low-income and middle-income countries such as India, China, Philippines and Thailand in Asia, ranging between 6% to 40% [19,20]. However, in high-income Asian countries, 5-year survival increased from 54.8% between 1993 to 1995 to 72.6% between 2006 to 2010 in South Korea [50], and it exceeds 60% in Singapore [27], due to early detection and better access to treatment. The increasing incidence of CRC in Asia and the improved prognosis in countries with well-developed health services such as South Korea and Singapore underscore the need to improve public healthcare infrastructures to support FOBT screening, colonoscopy, staging and treatment of CRC efficiently.

Improving access to surgery, radiotherapy and chemotherapy is critical for improving survival outcomes from several epithelial cancers such as head and neck, colorectal, stomach, ovarian and endometrial cancers. Oral visual inspection can lead to early diagnosis of oral cancer in users of tobacco and/or alcohol; endoscopy in high-risk individuals can lead to early diagnosis of oesophageal and stomach cancers. Regular clinical assessment using α-fetoprotein (AFP) and ultrasonography every 6 to 12 months in high-risk subjects with HBV and HCV infection, cirrhosis and alcohol drinkers should be considered in high-risk countries for the early diagnosis of hepatocellular carcinoma (HCC), when lesions are small and easier to treat with improved survival. Improving infrastructure and trained human resources in surgical techniques such as endoscopic mucosal and submucosal dissection [74-77], radiofrequency ablation and transcathetral arterial embolisation [78-81] should be considered for treatment of early oesophageal, stomach and liver cancers.

Lymphomas and leukaemia are major lymphoreticular neoplasms for which accurate diagnosis, typing, staging and optimum treatment are important strategies for disease control. In the case of non-Hodgkin lymphoma, which shows an increasing trend in incidence in Asia [8] assignment to subgroups such as indolent, aggressive, or acute leukaemia-like disease based on expected natural history is critical to guide both the urgency and nature of the primary treatment. Whereas a watch-and-wait policy of observation until the development of symptoms has been proposed for patients with indolent lymphomas, aggressive forms of lymphoma require immediate treatment [82-84]; after primary treatment, transformation to more aggressive types must be recognised and appropriately managed. Accurate histological subtyping and staging of Hodgkin’s lymphoma is critical for adequate management: patients with early stage disease are treated with short courses of combination chemotherapy followed by involved field radiotherapy, or with total nodal radiotherapy alone, whereas those with advanced stages are managed with long courses of combination chemotherapy. Successful treatment of acute lymphocytic leukaemia involves control of systemic and sanctuary site disease with combination chemotherapy, intrathecal chemotherapy or cranial radiotherapy. Treatment of acute myeloid leukaemia involves combination chemotherapy to control systemic and bone marrow disease. The high rate of treatment abandonment, due to unaffordability and patients living long distances from facilities, the inability to provide optimum treatment due to unavailability of medication, and treatment-related complications due to inadequate supportive care, limit the survival prospects of patients with lymphoreticular neoplasms [19,20,85,86]. Given the growing burden of these cancers, health service investments in accurate staging, immunohistochemistry, radiotherapy and essential chemotherapeutic drugs are critical to their improved control.

### Palliative care

Palliative care services are in transition and steadily growing, with a trend away from palliative care units towards palliative care teams and home care. Whereas it was once seen as the limited treatment of patients with terminal conditions, palliative care involving management of cancer pain and other symptoms due to advanced cancer is increasingly becoming integrated into mainstream treatment in all countries [87]. WHO proposed a three-step analgesic ‘ladder’ approach in 1986 as a framework for cancer pain relief in the following order: use of non-opioids (aspirin and paracetamol) and, then as necessary, mild opioids such as codeine, and then strong opioids such as morphine or tramadol or methadone until pain is relieved [88]. It recommended that drugs should be given round the clock every 2 to 6 h, rather than ‘on demand’, and adjuvant drugs may be added for effective pain relief. Lack of availability or underuse of opioids has limited its application in many Asian countries.

However, palliative care is more than pain relief and availability of oral morphine, since most patients with advanced cancer develop diverse symptoms undermining their quality of life. Relief of specific symptoms by judicious use of debulking surgery, radiotherapy and chemotherapy are particularly important: pain relief from bone and brain metastases, preventing impending paraplegia from spine metastases by palliative radiotherapy, and relief of symptoms of superior mediastinal obstruction by palliative chemotherapy or radiotherapy are some specific examples. The availability of palliative care services across Asian countries is widely variable and sustained development of these services have evolved in few high-income, and high-middle-income countries such as Japan and Turkey and in certain provinces in India such as Kerala [89-92]. Major barriers for the development of palliative care services in Asian countries include opioid phobia among professionals and narcotic control agencies, inadequate training programmes, lack of awareness and lack of understanding of palliative care needs at professional, policy and public levels, and lack of resources and of integration into mainstream health services and education. Palliative care education of general medical practitioners and nurses and development of home care are critically important to provide basic palliative care in several Asian countries.

### Improving cancer health services: who is responsible?

The key components of a well-functioning health system that provides equitable access to cancer prevention and control services are given in Table 5[18].

Adequate development of the various components of cancer health services given in Table 5 requires national political commitment to reform and invest in a phased manner. National governments are principally responsible for planning and implementing national cancer plans in a time-bound manner, linked with committed budget lines.

Carefully planned public/voluntary sector/private sector partnerships and phased development of healthcare financing involving government-sponsored social security schemes, universal health coverage and industrial sector and alignment of donor funds to national planning can lead to development cancer health services in LMICs in due course. Healthcare services in most Asian LMICs need to undergo a far-reaching reform process leading to radical changes in the provision and financing of healthcare services, as has been the case in countries such as Thailand and Turkey through the Health Transformation Program [93,94]. With the exception of Brunei, Japan, Korea, Malaysia, Singapore, Taiwan, Turkey and Thailand, most healthcare systems in Central East and South Asia provide very little financial risk protection and the role of public prepaid systems such as social health insurance is minimal, and catastrophic out-of-pocket payment is a major source of financing [94]. The governments in many Asian LMICs will need to double, triple, or quadruple their annual investment in healthcare infrastructure to improve healthcare services. Health service financing through social security schemes covering vulnerable segments of the population, schemes financed through employers, and employees and general health insurance schemes and adaptive pricing strategies for essential diagnostics, drugs and treatments can improve access to essential cancer diagnosis and treatment services, as has been shown in Thailand and Turkey [93,94].

Thailand investing 4.1% of GDP in healthcare (which is less than half of the OECD average of 9.9%) has achieved Universal Health Coverage (UHC) since 2002 (formerly known as the 30 Baht scheme to have preventive care and to treat all diseases including cancer in which the participant pays US$1 to enter healthcare services for a health problem), representing a source of inspiration for other LMICs in Asia. Currently countries such as Bangladesh, India, Philippines, Indonesia and Vietnam are working towards developing UHC.

## Conclusions

The above description of existing and emerging cancer patterns in Asia calls for a wide variety of interventions to reduce the burden of disease. This includes balanced investments in awareness and educational efforts targeting the general population and primary care practitioners, focused primary prevention efforts such as HBV vaccination, HPV vaccination, tobacco control measures and promotion of physical activity and healthy diets, vertical investment in strengthening cancer healthcare infrastructure and in augmenting trained human resources to provide cancer prevention, early detection, treatment and follow-up as well as palliative care services. Cancer healthcare services are poorly developed and organised in vast regions of Asia, particularly in rural areas of large countries such as China, India, Indonesia, Pakistan, Philippines and Bangladesh and in low-income countries such as Nepal, Laos, Cambodia, and Yemen, among others. Early detection and treatment of common cancers result in considerable savings in treatment costs; diagnosis of advanced stages of breast, colon, stomach, liver, lung and thyroid cancers was associated with 1.8 to 2.5-fold higher costs than early cancers in South Korea [95].

Awareness of the symptoms and signs of common cancers, empowering primary care practitioners in clinical suspicion and appropriate referral of patients with suspected cancer and early clinical diagnosis among patients who are symptomatic is a universally important early detection approach throughout Asia. However, introduction of organised screening programmes should be undertaken carefully and in a phased manner only when the organisation, resources and infrastructure within the wider public health infrastructure is adequate to support the demands for screening and expectations of clients. There is a great need to optimally improve the diagnostic infrastructure, comprising pathology, imaging and endoscopy services, in many regions. The capacity to provide optimum surgery, radiotherapy and chemotherapy and hormonal therapy services in public health services in many countries is far from satisfactory, and needs to be augmented in a phased manner. Development of capacity and resources to provide minimally invasive surgery, such as endoscopic resection for early digestive tract malignancies, requires particular attention. National and regional policies, uniform diagnosis and treatment policies for common cancers will improve the quality and cost effectiveness of care and will avoid overtreatment and use of inappropriate expensive treatment regimes, particularly chemotherapy. The need to provide basic essential chemotherapy and hormone therapy to treat common forms of cancers in public health services is clearly more important than using expensive targeted therapies at the expense of more essential drugs. Development of a set of minimal health system performance indicators covering prevention, screening, diagnosis, treatment, palliative care, patient experience and research can contribute to improving cancer health services in all Asian countries. The above initiatives require political commitment and the recognition of the fact that cancer is an important public health problem in Asia.

## Abbreviations

AFB: Aflatoxin B1; AFP: α-fetoprotein; ASR W: Age-standardised rate (standardised to world standard population); CBE: Clinical breast examination; CIN: Cervical intraepithelial neoplasia; CRC: Colorectal cancer; CT: Computed tomography; DALY: Disability-adjusted life-years; FNAC: Fine needle aspiration cytology; FOBT: Faecal occult blood testing; GDP: Gross domestic product; GNI: Gross national income; HBV: Hepatitis B virus; HCC: Hepatocellular carcinoma; HCV: Hepatitis C virus; HDI: Human development index; HL: Hodgkin’s lymphoma; Hong Kong SAR: Hong Kong Special Administrative Region; HPV: Human papillomavirus; LEEP: Loop electrosurgical excision procedure; LMICs: Low- and middle-income countries; Macao SAR: Macao Special Administrative Region; NHL: Non-Hodgkin lymphoma; PSA: Prostate-specific antigen; UHC: Universal health coverage; VIA: Visual inspection with acetic acid.

## Competing interests

The authors declare that they have no competing interests.

## Authors’ contributions

RS wrote the first draft of the manuscript, to which KR and YQ made contributions. Subsequent drafts were revised by all authors. All authors read and approved the final manuscript.
